# Thermally Stable Silver Cathode Covered by Samaria-Doped Ceria for Low-Temperature Solid Oxide Fuel Cells

**DOI:** 10.3390/nano14070561

**Published:** 2024-03-22

**Authors:** Davin Jeong, Gieun Jang, Soonwook Hong

**Affiliations:** Department of Mechanical Engineering, Chonnam National University, 77 Yongbong-ro, Buk-gu, Gwangju 61186, Republic of Korea; djeong@jnu.ac.kr (D.J.); word8820@jnu.ac.kr (G.J.)

**Keywords:** silver, samaria-doped ceria, overlayer, solid oxide fuel cell, thermal stability, oxygen reduction reaction

## Abstract

Samaria-doped ceria (SDC) overlayers were deposited on Ag cathodes by sputtering. The SDC sputtering time was varied to investigate the properties of the Ag–SDC overlayer cathode-coated fuel cells depending on the thickness of the SDC overlayers. Among the fabricated fuel cells, Ag with a 10-nm-thick SDC overlayer (Ag-SDC10) cathode-coated fuel cell exhibited the highest peak power density of 6.587 mW/cm^2^ at 450 °C, showing higher performance than a pristine Pt-coated fuel cell. Moreover, electrochemical impedance spectroscopy revealed that the Ag-SDC10 cathode-coated fuel cell significantly mitigated polarization loss originating from enhanced oxygen reduction reaction kinetics compared to the pristine Ag-coated fuel cell.

## 1. Introduction

Solid oxide fuel cells (SOFCs) are considered one of the most promising energy-conversion devices because of their high energy-conversion efficiency, fuel flexibility, and no emission of pollutants [1,2]. An SOFC is typically operated in the temperature range of 800 °C–1000 °C, which can cause some issues such as thermal degradation of catalysts and difficulty in material selection [3]. These thermal issues have been regarded as obstacles to the commercialization of SOFCs. Accordingly, many researchers have attempted to reduce the operating temperature of SOFCs to the range of 400 °C–600 °C, which can mitigate the thermal issues [4,5,6,7]. These low-temperature SOFCs (LT-SOFCs) have thus been extensively studied in the past few decades as a practical means for the commercialization of SOFCs [8]. However, LT-SOFCs still suffer from performance degradation due to increased ohmic and polarization losses at reduced operating temperatures. It is generally accepted that ohmic and polarization losses are functions of temperature, and the reduced operating temperature of SOFCs adversely affects their performance [9]. Therefore, thin-film fabrication processes such as physical vapor deposition have been developed to fabricate thin electrolytes and electrodes with thicknesses less than 1 μm to reduce ohmic losses in LT-SOFCs. These techniques have successfully demonstrated that thin films of electrolyte and electrodes can provide reduced electronic and ionic conduction pathways, resulting in the mitigation of ohmic losses in LT-SOFCs [10,11,12].

Another concerning issue is polarization loss, which still prevails at reduced operating temperatures. The main reason for polarization losses in LT-SOFCs is a sluggish oxygen reduction reaction (ORR) [13]. Consequently, many researchers have attempted to enhance ORR kinetics by adopting novel approaches such as a cathodic functional layer and mixed ionic–electronic conductor [14,15,16,17,18]. Although several approaches have been suggested, highly active noble metals such as platinum (Pt) have also been utilized owing to their exceptional catalytic activity, which can reduce polarization losses in the low-temperature region [19]. A Pt-based cathode has normally been used as a porous structure to increase the catalytically active area. However, this porous structure is vulnerable to structural changes even at alleviated temperatures because agglomeration easily occurs, reducing the high surface energy of the porous structure [20,21]. Furthermore, Pt is well-known for its high cost and scarcity among noble metals; therefore, there have been several investigations to find alternative materials [22,23]. Silver (Ag), as an alternative to Pt, has drawn attention because it has not only shown comparable ORR kinetics but is also cheaper than Pt [24,25,26,27,28]. Strategies using Ag as a cathode have been extensively studied to enhance ORR kinetics in LT-SOFCs by using oxide ionic conductors. Choi et al. suggested that fuel cells employing Ag cathodes with scandia-stabilized zirconia could outperform Pt-coated fuel cells, indicating the versatile application of Ag cathodes for LT-SOFCs [27]. Lee et al. also demonstrated that an Ag cathode infiltrated with samaria-doped ceria (SDC) could successfully increase ORR kinetics by increasing the triple-phase boundary (TPB), which is the active site of electrochemical reactions and physical meeting point between Ag, SDC, and oxygen gas [28]. In addition to the abovementioned reports, many studies have concluded that the Ag cathode requires a passivation layer to prevent surface oxidation and agglomeration [29,30].

Oxygen-ion-conducting materials have been utilized to cover the Ag cathode as a passivation layer because these materials normally have high thermal stability. Therefore, ceria-based materials such as gadolinia-doped ceria and SDC have been considered candidates for passivate Ag cathodes owing to their higher oxygen ion conductivity compared with zirconia-based materials [31]. However, the thickness of the passivation layer for the Ag cathode is a key factor in preventing the thermal agglomeration of the Ag cathode as well as for enhancing ORR kinetics by increasing the TPB density at the interface between Ag and the passivation layer. A thicker oxygen-ion-conducting layer on the Ag cathode may afford higher thermal stability and can also block the electrochemically reactive surface of the Ag cathode.

**Figure 1:**
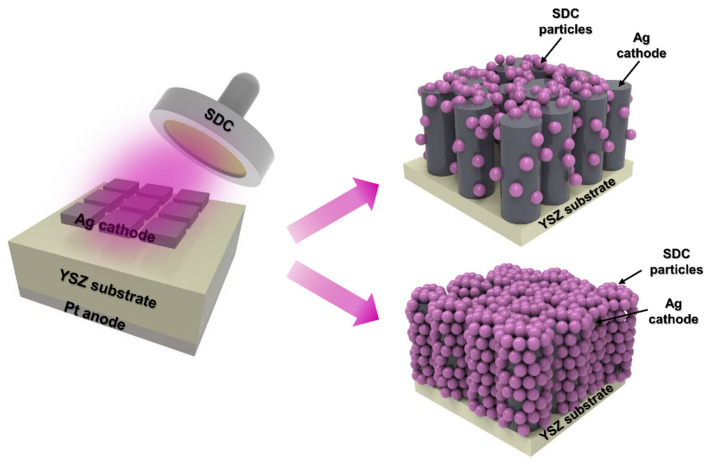
Schematic of the sputtering process used to fabricate LT-SOFCs with Ag–SDC overlayered cathodes.

In this study, we successfully demonstrated an approach to protect the Ag cathode from agglomeration with an SDC overlayer via sputtering (Figure 1). The concept of an overlayer, which is a passivation layer with SDC on an Ag cathode, revealed that a passivated Ag cathode with SDC showed increased thermal stability as well as ORR kinetics. However, a large amount of SDC particles can induce a reduction in the number of sites for oxygen gas diffusion, resulting in decreased performance of fuel cells. Thus, the optimal SDC thicknesses were investigated by controlling the thickness of SDC with sputter deposition time. All the fuel cells coated with SDC overlayers of different thicknesses were analyzed through electrochemical measurements to determine the optimized thickness of the SDC overlayer. The presence of the SDC overlayer clearly increased the thermal stability of the Ag cathode while increasing the performance of fuel cells, with enhanced TPB sites at the interlayer between Ag and SDC by catalyzing ORR kinetics, outperforming the Pt cathode-coated fuel cell. The findings of this study will facilitate the fabrication of LT-SOFC at low cost and can potentially be extended to other electrochemical applications requiring thermal stability.

## 2. Materials and Methods

### 2.1. Sample Preparation

Single-crystalline silicon (100) substrates with dimensions of 1 cm × 1 cm × 500 μm were used to study the morphologies and properties of the fabricated SDC overlayers. The LT-SOFCs were fabricated on commercially available 8-mol% yttria-stabilized zirconia (YSZ) substrates as electrolyte-supporting structures with dimensions of 1 cm × 1 cm × 200 μm (MTI Corporation, Richmond, CA, USA). Both silicon and YSZ substrates were cleaned ultrasonically in the following sequence: acetone, isopropyl alcohol, and deionized water for 30 s to remove surface contaminants. After cleaning the substrates, they were placed on a hotplate for 5 min to dry the remaining water. A magnetron sputtering system (NEO, JA Innovation, Yongin, Republic of Korea) was employed to fabricate Ag, SDC, and Pt for cathodes, cathode overlayers, and anodes, respectively. During the sputtering process, substrates were rotated at a fixed value of 4.3 rpm to ensure uniform deposition. A silver sputtering target (RND Korea Corp, Gwangmyeong, Republic of Korea) of 99.99% purity with a 2-inch diameter was used to fabricate the Ag cathodes with dimensions of 1 mm × 1 mm with a physical shadow mask. SDC overlayers were deposited on the Ag cathodes using a 2-inch diameter SDC target composed of Sm_0.15_Ce_0.85_O_2−x_ (Taewon Scientific Co., Ltd., Seoul, Republic of Korea). In addition, we fabricated a fuel cell with Pt coated on both anode and cathode as a control fuel cell to directly compare its electrochemical performance with that of the Ag–SDC coated fuel cells.

The Ag cathodes were deposited on YSZ substrates at a radio frequency (RF) power of 100 W, an argon flow rate of 20 sccm, and working pressure of 10 Pa. The SDC overlayers were sequentially fabricated on Ag cathodes at an RF power of 50 W, argon flow rate of 40 sccm, and working pressure of 1 Pa. Before the deposition process, the surfaces of both Ag and SDC sputtering targets were pre-sputtered for 10 and 15 min, respectively, to guarantee the properties and reproducibility of the fabricated thin films by removing contaminated target surfaces. Further, Pt anodes were deposited on another side of the YSZ substrates with a DC power of 100 W, argon flow rate of 40 sccm, and working pressure of 10 Pa. To fabricate fuel cells with SDC overlayers of different thicknesses, the sputtering time was varied for 8, 15, and 47 min to prepare 5-, 10-, and 30-nm-thick SDC overlayers, respectively; the thickness of Ag cathodes was fixed at ~200 nm. Hereinafter, the Ag cathode without an SDC overlayer and 5-, 10-, and 30-nm-thick SDC overlayers on Ag cathodes are denoted as pristine Ag cathode, Ag-SDC5, Ag-SDC10, and Ag-SDC30, respectively, in figures and tables. The Pt anode- and cathode-coated fuel cell was fabricated under the same sputtering conditions of Pt anode deposition as described above.

### 2.2. Fuel Cell Characterization

The surface morphologies and chemical compositions of the SDC overlayer-coated Ag cathodes were characterized using scanning electron microscopy (SEM, S-4700, Hitachi, Tokyo, Japan) and X-ray photoelectron spectroscopy (XPS, K-Alpha, Thermo Fisher Scientific, Waltham, MA, USA), respectively. Before the XPS analysis, ion etching was conducted for 3 min to remove carbon contaminations on the surfaces. The obtained XPS results were refined using the Shirley method to subtract the baseline and then deconvoluted using the Gaussian model. To further analyze the morphologies of the SDC overlayer-deposited Ag cathode, transmission electron microscopy (TEM, JEM-2100F, JEOL Ltd., Tokyo, Japan) and energy dispersive spectroscopy (EDS) were employed. The TEM specimen was prepared by ion milling for electron transparency (Ion Milling System, PIPS691, Gatan Inc., Pleastanton, CA, USA). A hole in the center of the sample was fabricated with 5 keV biased argon ion beams with an incident angle of 6°. The bias of argon beams was reduced to 500 eV at a final process for a clean sample surface. The electrochemical performances of the fabricated fuel cells with SDC overlayer-coated Ag cathodes were measured using an in-house-fabricated probing system with heat applied using a heat controller to maintain a temperature of 450 °C and purity of 99.999%. Hydrogen was applied to a Pt anode with a flow rate of 20 sccm. In addition, a gold ring was utilized to ensure gas tightness between the fuel cell and substrate holder. Linear sweep voltammetry (LSV) was used from open circuit voltage to 0.2 V to obtain the current–voltage behavior (Interface 1010E, Gamry Instruments Co., Ltd., Warminster, PA, USA). Electrochemical impedance spectroscopy (EIS) was conducted using the abovementioned apparatus in the range of 1 MHz–1 Hz while maintaining a temperature of 450 °C and a DC bias voltage of 0.8 V. The results of EIS spectra were fitted using equivalent circuit modeling with the Gamry software version 7.9.0 (Gamry Instruments Inc., Echem Analyst). Furthermore, the thermal stabilities of the fuel cells were measured at 450 °C and 0.5 V for 20 h using chronoamperometry.

## 3. Results and Discussion

**Figure 2:**
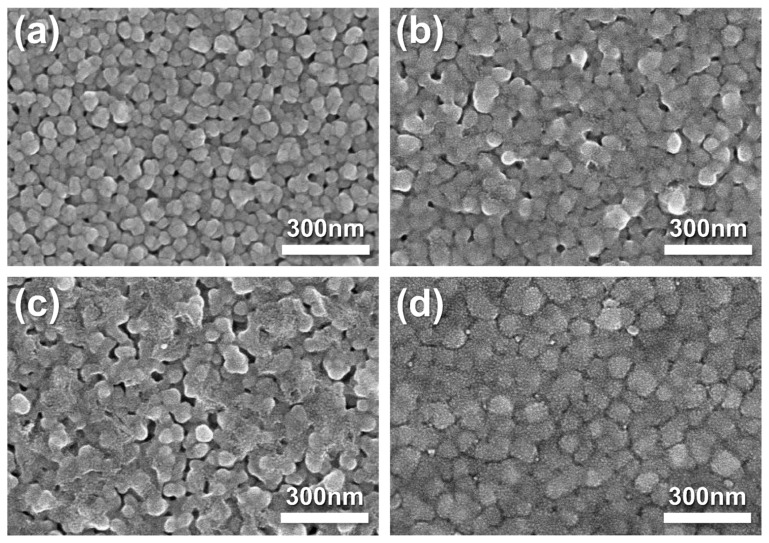
Top-view SEM images of as-deposited (**a**) pristine Ag, (**b**) Ag-SDC5, (**c**) Ag-SDC10, and (**d**) Ag-SDC30.

The morphologies of the fabricated pristine Ag cathode and Ag–SDC overlayers were observed using SEM (Figure 2). Pristine Ag cathode was found to have a porous morphology. A fuel cell with a porous electrode is generally known to have a higher TPB density than a dense electrode [25,29,32]. The Ag–SDC cathodes overlayered with SDC overlayers of different thicknesses are shown in Figure 2b–d. The SEM images revealed that the SDC overlayers were deposited on the porous Ag cathodes and exhibited slightly different surface morphologies compared to the pristine Ag cathode. With the increasing thickness of the SDC overlayer deposited on the Ag cathodes, denser morphologies were observed. These SDC overlayers could preserve the porous morphologies of Ag cathodes, avoiding thermal agglomeration. Because the Ag cathode had a porous structure, SDC particles not only covered the surface of Ag cathodes but also penetrated the Ag cathodes, leading to increased TPB density as illustrated in Figure 1. Thus, the Ag–SDC overlayer-coated fuel cells may enhance the electrochemical performance compared to the pristine Ag cathode-coated fuel cell owing to increased ORR kinetics caused by an increase in the TPB density.

**Figure 3:**
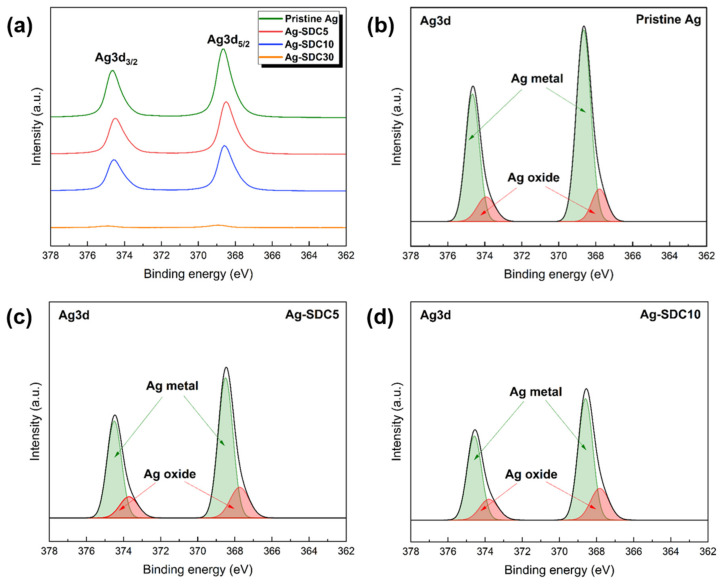
XPS spectra of Ag3d. (**a**) Ag3d spectra of fabricated Ag cathodes. XPS spectral fitting of Ag metal and Ag oxide for (**b**) pristine Ag, (**c**) Ag-SDC5, and (**d**) Ag-SDC10.

The chemical compositions and oxidation states of the pristine Ag cathode and Ag–SDC overlayers were analyzed using XPS. The results revealed the existence of elemental Ag, O, Sm, Ce, and C on the surface of the Ag–SDC overlayers as given in Table 1. In the pristine Ag cathode, 9.02 at% of oxygen, which came mainly from the oxygen in the atmosphere (i.e., native oxide), was detected. As the thickness of the SDC overlayers increased, the atomic percentage of Ag decreased while those of both Sm and Ce, which are components of the SDC, increased. The 30-nm-thick SDC overlayer almost covered the surface of the Ag cathode so that only 3.33 at% of elemental Ag was observed in Ag-SDC30. The XPS spectra of Ag3d for the fabricated cathodes were investigated to further study the oxidation states, as shown in Figure 3. All the peaks associated with Ag3d were observed near the binding energies of 374.7 and 368.7 eV. However, the XPS spectra of Ag3d for the Ag-SDC30 cathode were barely visible because of the lowered atomic percentage of elemental Ag, as indicated in Table 1. To evaluate the oxidation state of Ag, XPS spectral fitting was performed to deconvolute the spectra into Ag metal and Ag oxide peaks (Figure 3b–d). The peaks of Ag3d for the pristine Ag cathode indicated the highest intensity; the presence of native oxide of Ag was also observed in the Ag3d spectra (Figure 3b). Compared to the pristine Ag cathode, Ag–SDC overlayers exhibited almost similar amounts of Ag oxidation states. It was confirmed that the peaks of Ag3d for Ag-SDC5 and Ag-SDC10 had lower intensities with an increase in SDC content on the Ag surface.

**Figure 4:**
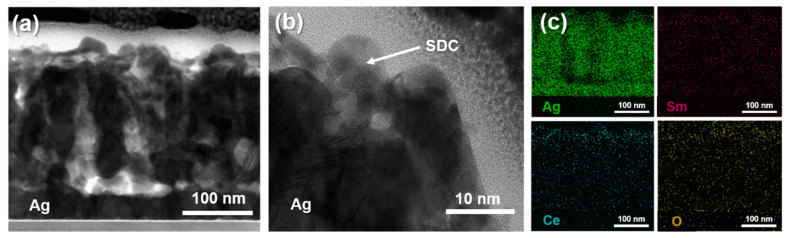
Cross-sectional TEM images of (**a**,**b**) Ag-SDC10 and (**c**) EDS elemental mapping results of Ag-SDC10.

To further investigate the morphology and quality of the fabricated Ag cathode with an SDC overlayer, TEM and EDS elemental mapping results were utilized. Figure 4a,b presents representative cross-sectional TEM images with different magnifications to show the morphology of the Ag-SDC10 cathode. Because the thickness of the SDC was too thin, it was difficult to determine whether the Ag cathode was fully covered with the SDC overlayer. However, the results of EDS elemental mapping clearly indicated that the SDC overlayer successfully covered the Ag cathode, penetrating the interior of the porous Ag cathode as well (Figure 4c). Although one can claim that a 10-nm-thick SDC overlayer appears to not fully cover the porous Ag cathode, some studies demonstrated that ultrathin overlayers successfully mitigated the agglomeration of porous cathodes [33,34,35]. Accordingly, we suggest that a 10-nm-thick SDC overlayer could increase the thermal stability of the Ag cathode. Considering the TEM and EDS results, LT-SOFCs with Ag–SDC overlayered cathodes are expected to enhance thermal stability and performance compared to pristine Ag cathode-coated LT-SOFCs.

**Figure 5:**
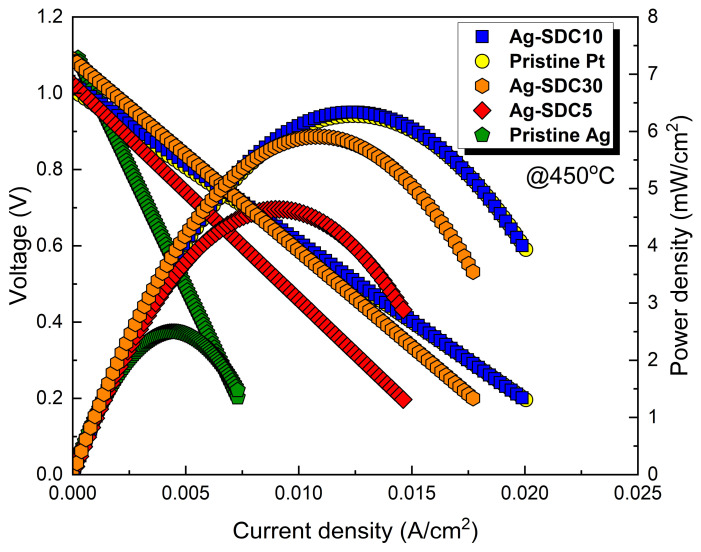
Current–voltage behavior of fuel cells with pristine Pt, pristine Ag, and Ag-SDC-overlayered cathodes measured at 450 °C.

To investigate the performances of the fabricated LT-SOFCs, current–voltage behaviors were measured at the operating temperature of 450 °C as shown in Figure 5. We first evaluated Pt and Ag cathode-coated fuel cells, which were considered control fuel cells, to directly compare the electrochemical performance of Ag–SDC overlayer-coated fuel cells. The pristine Pt cathode-coated fuel cell showed a peak power density of 6.217 mW/cm^2^, almost twice that of the pristine Ag cathode-coated fuel cell, which showed a peak power density of 3.124 mW/cm^2^ at 450 °C. These results were attributed to the difference in catalytic activity between Pt and Ag. The fuel cells with Ag–SDC overlayered cathodes exhibited significantly enhanced performances compared to the pristine Ag cathode-coated fuel cell. The LT-SOFCs with Ag–SDC overlayered cathodes had a peak power density of 4.814, 6.587, and 5.843 mW/cm^2^ for Ag-SDC5, Ag-SDC10, and Ag-SDC30, respectively. All the fuel cells with Ag–SDC overlayered cathodes showed enhanced peak power densities compared to the pristine Ag cathode-coated fuel cell. However, Ag-SDC5- and Ag-SDC30-coated fuel cells showed degraded performances compared to Ag-SDC10-coated fuel cells. Notably, the Ag-SDC10-coated fuel cell exhibited a higher peak power density than the pristine Pt cathode-coated fuel cell. Thus, the optimized thickness of the SDC overlayer on the Ag cathode could increase the performance of the fuel cell, outperforming Pt in terms of catalytic activity.

**Figure 6:**
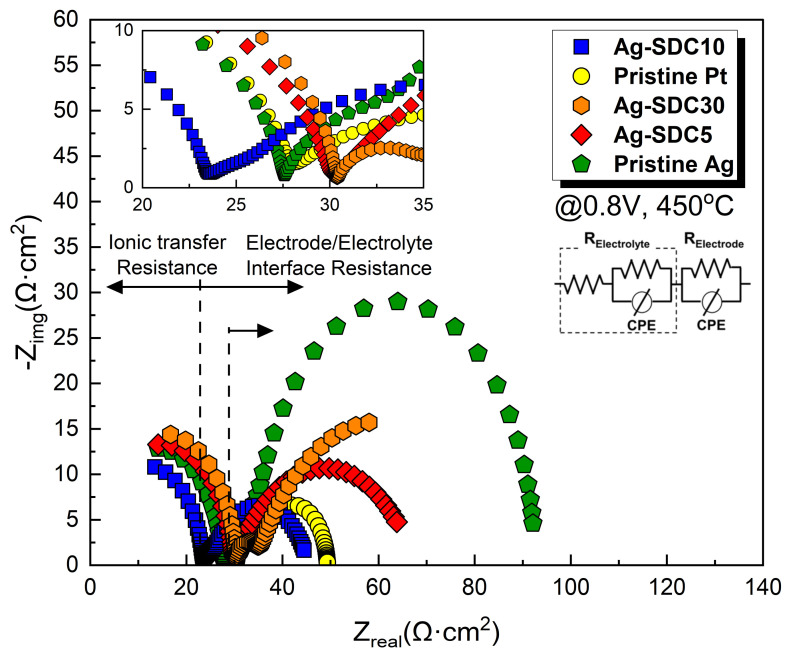
Electrochemical impedance spectra of fuel cells with pristine Pt, pristine Ag, and Ag–SDC overlayered cathodes measured at 450 °C under a DC bias voltage of 0.8 V.

EIS analysis was employed to determine the main factor influencing the performance enhancement of the LT-SOFCs. The EIS analysis was conducted at a DC bias voltage of 0.8 V, maintaining the fuel cell operating temperature at 450 °C (Figure 6). The obtained EIS results were fitted using an equivalent circuit model to confirm all resistors to related semicircles. The first semicircle of EIS spectra represents ohmic losses and the radius of the second semicircle denotes polarization losses [36]. A slight difference in terms of ohmic losses was observed among the fuel cells, while there was a significant variation in terms of polarization losses. Ag cathode-coated fuel cells showed higher polarization losses than Pt cathode-coated fuel cells. Moreover, the fuel cells with Ag–SDC-overlayered cathodes showed reduced polarization losses compared to Ag cathode-coated fuel cells. The Ag-SDC10-coated fuel cell exhibited the lowest polarization loss among the fabricated fuel cells. This result could be attributed to enhanced ORR kinetics due to the increase in TPB density, which was mainly caused by the SDC particles penetrating the Ag cathode as confirmed in Figure 4. However, Ag-SDC5- and Ag-SDC30-coated fuel cells revealed slightly increased polarization losses compared to Ag-SDC10-coated fuel cells. We speculate that Ag-SDC5- and Ag-SDC30-coated fuel cells had insufficient and excessive amounts of SDC particles on the Ag cathode, respectively. Thus, the fuel cell with Ag-SDC5 could not enhance the TPB density because of insufficient SDC content, while the Ag-SDC30 overlayer could almost cover the Ag cathode, resulting in decreased oxygen permeability of the cathode. These results directly influenced the performance of fuel cells in terms of ORR kinetics. Therefore, it can be concluded that the polarization losses in the Ag–SDC overlayered cathodes could be reduced by optimizing the thickness of the SDC overlayer.

**Figure 7:**
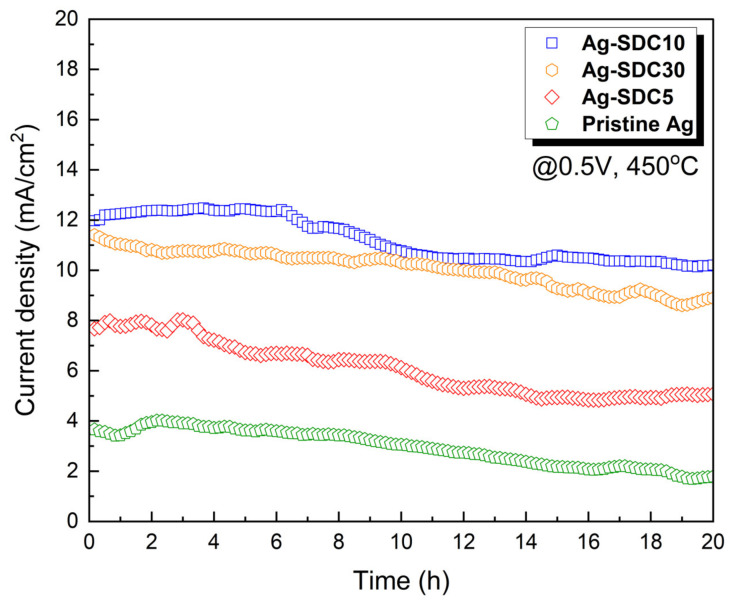
Thermal stability measurements of fuel cells with pristine Ag and Ag–SDC overlayered cathodes measured at 450 °C under a DC bias voltage of 0.5 V for 20 h.

To evaluate the thermal stability based on the thickness of SDC overlayers, chronoamperometry was conducted for 20 h at 450 °C under a DC bias voltage of 0.5 V (Figure 7). Ag-SDC10-coated fuel cells exhibited the highest current density during the measurement, followed by Ag-SDC30-, Ag-SDC5-, and pristine Ag-coated fuel cells with the same tendency as that shown in Figure 5. Ag-SDC30- and Ag-SDC10-coated fuel cells demonstrated less performance degradation during the measurement. However, Ag-SDC5 and pristine Ag underwent rapid degradation owing to the insufficient and uncovered surfaces of Ag cathodes, showing more than 30% and 50% degradation, respectively. Thus, it was demonstrated that the thicker the SDC overlayers deposited on the Ag cathodes, the higher the thermal stability obtained for the thermally vulnerable Ag cathode-coated fuel cells.

**Figure 8:**
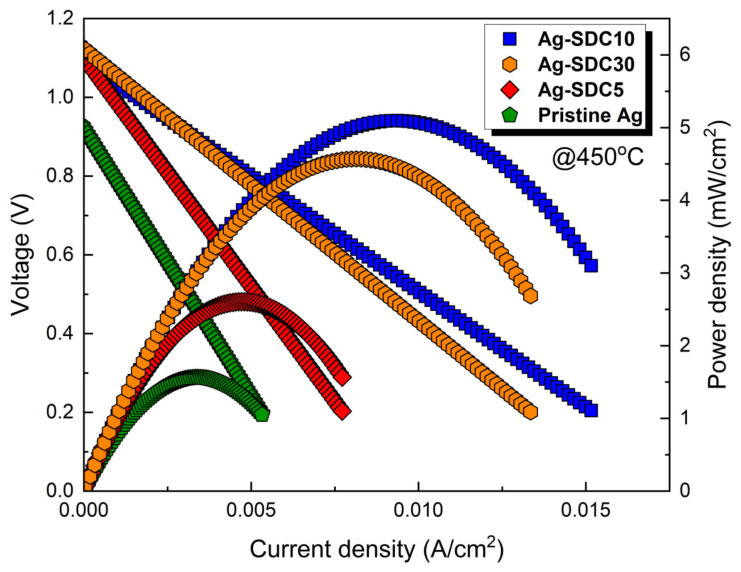
Current–voltage behavior of fuel cells with pristine Ag and Ag–SDC overlayered cathodes measured at 450 °C after thermal stability measurement.

Figure 8 shows the current–voltage behavior of the LT-SOFCs with pristine Ag and Ag–SDC overlayered cathodes after 20 h of thermal stability measurement. All the fuel cells showed decreased peak power densities compared to the previous current–voltage behavior (Figure 5). The Ag-SDC10-coated fuel cell had a peak power density of 5.089 mW/cm^2^, which was the highest among all fuel cells (4.507, 2.611, and 1.528 mW/cm^2^ for Ag-SDC30-, Ag-SDC5-, and pristine Ag-coated fuel cells, respectively). The fuel cells with Ag-SDC5 and pristine Ag exhibited significantly degraded peak power densities after chronoamperometry measurement, while the Ag-SDC30- and Ag-SDC10-coated fuel cells showed lower performance degradation. These results stem from severe degradations during the thermal stability measurement, as observed in Figure 7.

**Figure 9:**
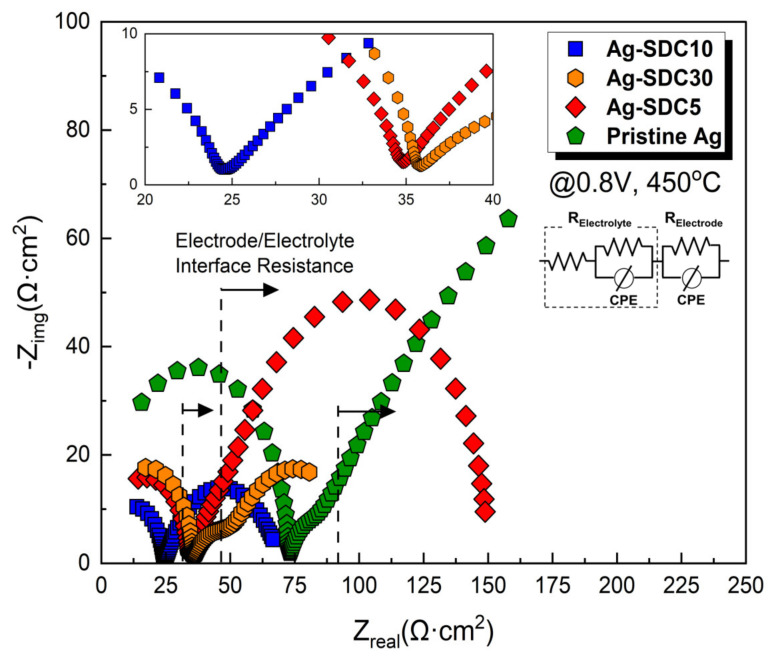
Electrochemical impedance spectra of fuel cells with pristine Ag and Ag–SDC-overlayered cathodes measured at 450 °C under a DC bias voltage of 0.8 V.

To further investigate the degraded behavior of the LT-SOFCs, an EIS analysis was conducted (Figure 9). The ohmic losses and polarization losses of all the fuel cells were found to increase after the chronoamperometry measurement. The increased ohmic losses could be attributed to the oxidation of Ag cathodes during the 20 h operation. Clearly, the oxidized Ag cathode reduced the percolation pathways, causing sluggish transportation of electrons. Thus, Ag cathode-coated fuel cells showed significantly increased ohmic losses due to the absence of SDC particles, while fuel cells with Ag–SDC overlayered cathodes indicated a minor increase in ohmic losses [30]. Furthermore, Ag cathode-coated fuel cells exhibited considerably increased polarization losses. This phenomenon was revealed by the thermal agglomeration of porous Ag cathodes following 20 h of exposure at 450 °C, resulting in decreased TPB density [29]. However, the fuel cells with Ag–SDC-overlayered cathodes revealed relatively mitigated polarization losses compared with Ag cathode-coated fuel cells owing to the presence of SDC overlayers.

**Figure 10:**
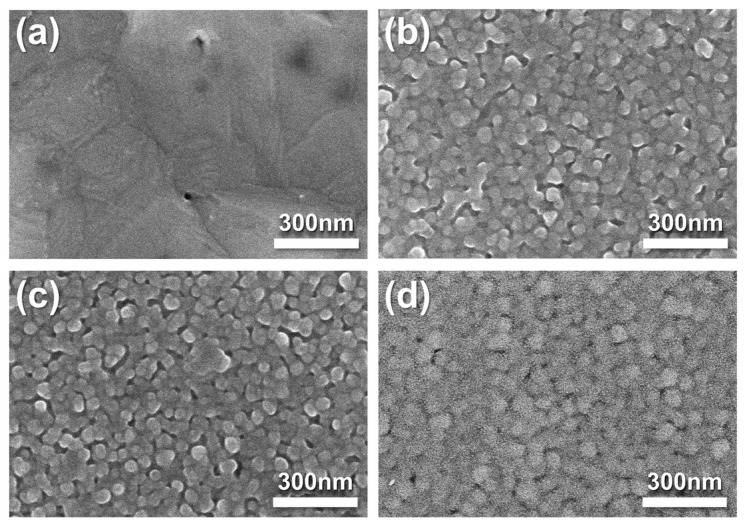
Top-view SEM images acquired after thermal stability measurement of fuel cells with (**a**) pristine Ag, (**b**) Ag-SDC5, (**c**) Ag-SDC10, and (**d**) Ag-SDC30.

SEM was utilized to observe the morphological changes in the fuel cells after thermal stability measurement. Figure 10a shows severe agglomeration in the pristine Ag cathode, which led to decreased ORR kinetics. By contrast, the SDC-overlayered Ag cathodes showed well-preserved morphologies, indicating greater thermal stability compared with pristine Ag cathodes (Figure 10). We confirmed that the SDC overlayers on the Ag cathode deposited by sputtering could successfully mitigate the thermal agglomeration of Ag cathodes and increase TPB density, affording the performance enhancement of LT-SOFCs.

## 4. Conclusions

In this study, Ag–SDC overlayer-coated fuel cells were fabricated by sputtering, and the material properties of the cathodes were evaluated. SDC, a thermally resistive ionic conductor, was deposited on Ag cathodes with different thicknesses by adjusting the sputtering time to prevent the thermal degradation of the Ag cathodes. The results of XPS and TEM-EDS elemental mapping confirmed that the SDC particles successfully covered and penetrated the porous Ag cathodes. The optimized Ag-SDC10-coated fuel cell showed a peak power density of 6.587 mW/cm^2^ at 450 °C, which was slightly higher than that of a pristine Pt-coated fuel cell. Furthermore, the EIS results indicated that the fuel cells with Ag–SDC-overlayered cathodes showed significantly reduced polarization losses because of enhanced ORR kinetics, which was caused by the increased TPB density. Furthermore, Ag-coated fuel cells with SDC overlayers demonstrated improved thermal stability compared to pristine Ag-coated fuel cells during 20 h operation. It was also confirmed that the SDC overlayers on Ag cathodes could prevent agglomeration. Thus, Ag–SDC overlayer-coated fuel cells outperformed pristine Ag-coated fuel cells. The results of this report suggest the possibility of replacing Pt with an Ag–SDC cathode material in LT-SOFCs by employing an SDC overlayer on the Ag cathode.

## Figures and Tables

**Table 1:** XPS analysis of surface compositions of as-deposited pristine Ag and Ag–SDC overlayers.

	Pristine Ag	Ag-SDC5	Ag-SDC10	Ag-SDC30
Ag3d (at%)	89.29	76.12	65.06	3.33
Ce3d (at%)	0	2.98	5.1	25
Sm3d (at%)	0	0.62	1.8	5.64
O1s (at%)	9.02	20.28	25.56	66.03
C1s (at%)	1.69	0	2.48	0

## Data Availability

Data are contained within the article.
